# The executive disruption model of tinnitus distress: Model validation in two independent datasets using factor score regression

**DOI:** 10.3389/fpsyg.2023.1006349

**Published:** 2023-02-10

**Authors:** Nathan A. Clarke, Michael A. Akeroyd, Helen Henshaw, Deborah A. Hall, Wan Najibah Wan Mohamad, Derek J. Hoare

**Affiliations:** ^1^Hearing Sciences, Mental Health and Clinical Neurosciences, University of Nottingham, Nottingham, United Kingdom; ^2^National Institute for Health and Care Research (NIHR) Biomedical Research Centre, Nottingham, United Kingdom; ^3^School of Social Sciences, Heriot-Watt University Malaysia, Putrajaya, Malaysia; ^4^Audiology Programme, School of Health Sciences, Universiti Sains Malaysia, Kubang Kerian, Malaysia

**Keywords:** tinnitus, cognition, tinnitus severity, tinnitus distress, executive function

## Abstract

This study presents the executive disruption model (EDM) of tinnitus distress and subsequently validates it statistically using two independent datasets (the Construction Dataset: *n* = 96 and the Validation Dataset: *n* = 200). The conceptual EDM was first operationalised as a structural causal model (construction phase). Then multiple regression was used to examine the effect of executive functioning on tinnitus-related distress (validation phase), adjusting for the additional contributions of hearing threshold and psychological distress. For both datasets, executive functioning negatively predicted tinnitus distress score by a similar amount (the Construction Dataset: *β* = −3.50, *p* = 0.13 and the Validation Dataset: *β* = −3.71, *p* = 0.02). Theoretical implications and applications of the EDM are subsequently discussed; these include the predictive nature of executive functioning in the development of distressing tinnitus, and the clinical utility of the EDM.

## Introduction

Subjective tinnitus is the perception of sound in the absence of corresponding sound energy external to the observer. In a recent Pan-European study, the prevalence of tinnitus was estimated to be 8.7–28.3%; the study estimated one in 15 people to have ‘bothersome tinnitus’ and one in 100 people to have ‘severe tinnitus’ (Biswas et al., 2022). ‘Severe tinnitus’ and ‘bothersome tinnitus’ are frequently used synonymously with ‘distressing tinnitus’, with the latter being the preferred term in the remainder of this article (De Ridder et al., 2021).

Distressing tinnitus has been associated with various negative outcomes including increased anxiety, depression, stress and disrupted sleep (Mohan et al., 2022). Concentration difficulties measured *via* self-reported measures (i.e. multi-item questionnaires), as well as tasks assessing performance in various cognitive domains, have also been associated with subjective tinnitus including processing speed, general short-term memory, general learning and retrieval and executive functioning (Clarke et al., 2020). Although concentration difficulties can be self-reported, performance on tasks of executive functioning may be considered an objective operationalisation of concentration ability in the context of tinnitus.

Executive functions have been analogised as cognitive ‘control systems’ and enable people to engage in complex goal-directed behaviour (Duncan, 2010; Diamond, 2013). Conceptualisations of executive functioning have largely been informed by neuropsychological observations of patients with frontal lobe damage as well as functional neuroimaging data in healthy participants. Different theoretical models have been proposed but they share a common view that executive functioning controls and manages other systems, abilities and processes. Such coordination requires a number of higher-level cognitive processes such as attention, shifting/cognitive flexibility, inhibitory control, initiation, organisation, planning, self-monitoring and working memory. There is substantial overlap with fluid intelligence, viewed as an emergent ‘higher-order’ executive function, enabling reasoning, planning and monitoring (Diamond, 2013). Importantly, performance-based tasks that measure executive functioning are confounded by ‘task impurity’ (Rabbitt, 2004), whereby all measure non-specific executive function to some extent.

Self-reported concentration difficulties are common in people experiencing distressing tinnitus, and executive functioning is likely to be a contributing factor; synthesis of the literature of associations between tinnitus and cognitive performance across a variety of domains has shown strongest associations between tinnitus and executive functioning (Clarke et al., 2020). Furthermore, research in chronic pain (with which tinnitus is often compared), suggests that executive functioning may predict tinnitus distress. Attal et al. (2014) prospectively sampled participants who underwent cognitive performance testing prior to surgery. They reported that performance on executive functioning tasks was predictive of which participants subsequently developed chronic pain after undergoing surgery, suggesting that executive functioning had a causal role in subsequent pain perception.

A frequent conclusion of studies investigating the relationship between tinnitus and cognition is that poorer cognitive performance is caused by ‘severe’ tinnitus (Mohamad et al., 2016). Here, an alternative theory and accompanying conceptual model are proposed to explain this association, suggesting this direction of causality is reversed, and that executive functioning plays a central causal role in distressing tinnitus. In the remainder of this article, the conceptual model—the executive disruption model (EDM)—is explicated and translated into a causal modelling framework (McElreath, 2016; Pearl et al., 2016). Results from empirical investigations are then presented that provide preliminary validation of the EDM, by applying the causal model to obtain an estimate of the effect of executive functioning on tinnitus distress in two independent datasets.

## Executive disruption model of tinnitus distress

### Rationale

The development of causal models of tinnitus distress has been limited (Schmidt et al., 2014), with some notable exceptions (Cima et al., 2011; McKenna et al., 2014; Handscomb et al., 2019). Like these models, the EDM makes a fundamental distinction between tinnitus distress and tinnitus percept. The *loudness* of a tinnitus percept is conceptualised as a sound attribute that has equivalence to that of an external sound stimulus such as a tone or noise; while tinnitus *distress* is a related but separable reaction to the percept (Hallam et al., 1984; Spankovich, 2021; Mohan et al., 2022).

**Figure 1:**
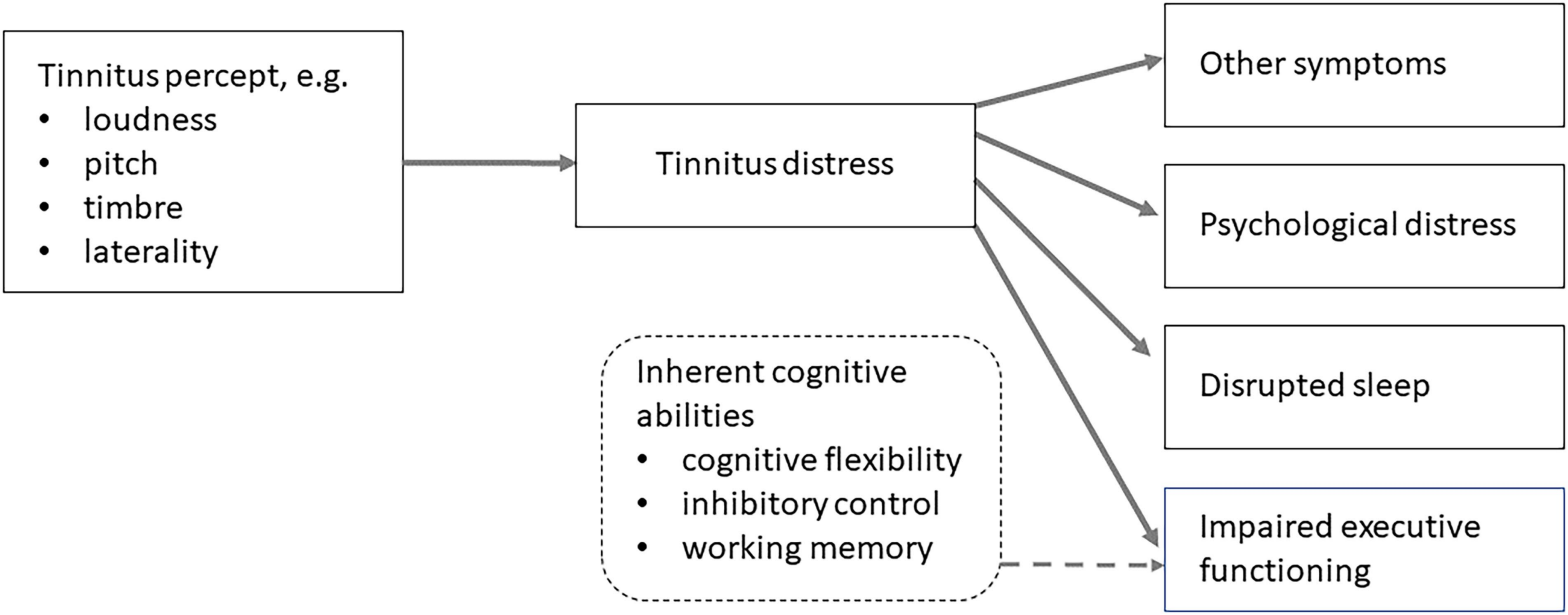
Path diagram illustrating how the tinnitus percept (e.g. loudness) can lead to a cascade of tinnitus distress and subsequent symptoms such as general psychological distress, impaired cognition and executive functioning and disrupted sleep. Dashed box and arrow indicates the cognitive processes that comprise executive functioning which is a latent construct.

Although the precise nature of the relationship between tinnitus loudness and distress is debated, Hallam et al. (1984) has noted a common assumption within the tinnitus literature is that ‘the most annoying aspect of tinnitus is its loudness’. This is likely still a prevalent assumption. A consequence of this ‘loud sounds are distracting’ assumption, appears to have spawned a further assumption that loud tinnitus disrupts executive functioning (Heeren et al., 2014; Tegg-Quinn et al., 2016). There is an intuitive appeal to such conceptualisations. Moreover, the most common measures of tinnitus distress are self-reported, and it is important to note that these measures also tacitly assume that the percept is *causing* the subsequent rating. For example, every question on the Tinnitus Functional Index (Meikle et al., 2012) contains this assumption by asking: ‘*Over the past week how often did your tinnitus interfer*e with your ability to concentrate/think clearly/focus your attention on other things besides your tinnitus?’. The fundamental nature of this relationship as a causal assumption is depicted in Figure 1.

**Figure 2:**
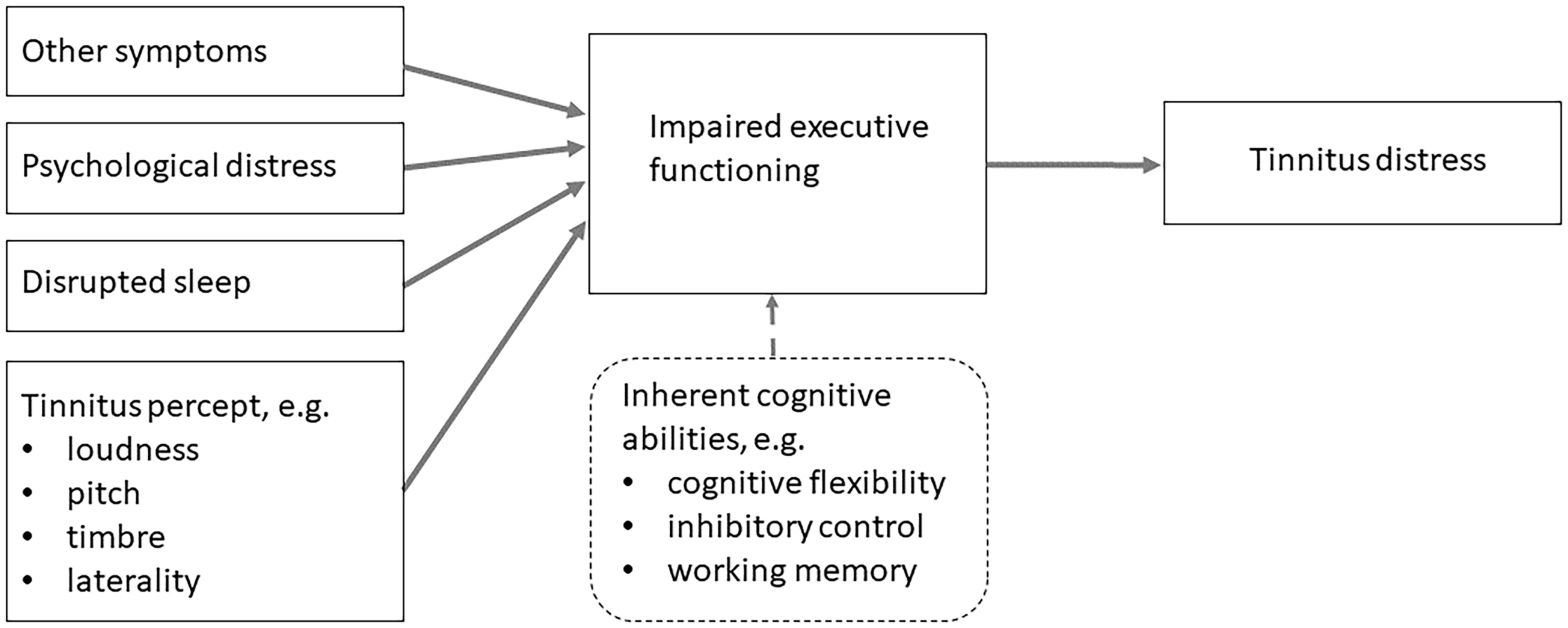
The executive disruption model (EDM) of tinnitus distress posits that the impact of the tinnitus percept (e.g. loudness) on tinnitus distress is moderated by executive functioning.

Here, an alternative causal mechanism is proposed, where inherent and disrupted executive abilities facilitate intrusion from the tinnitus percept (Figure 2). In this conception, established associations within the tinnitus literature that are also known to disrupt executive functioning (e.g. psychological distress and disrupted sleep) are causal agents contributing to tinnitus distress through increasing the intrusiveness off the percept. Andersson and McKenna (2006) first suggested that cognitive factors, such as ‘working memory deficits might worsen the effects of tinnitus, while still not being caused by tinnitus’. The idea that tinnitus distress may be worsened through other causes is a crucial insight. The insight has subsequently been elaborated and developed into a scientifically testable model; (McKenna et al., 2014). Handscomb et al. (2019) used structural equation modelling to show empirical support for the model.

The conceptual model proposed in this article - the EDM—represents a synthesis of a systematic review and meta-analysis of the literature between subjective tinnitus and cognitive performance, observations from the wider literature, behavioural research into the relationship between tinnitus distress, concentration difficulties and cognitive performance, discussions with people with tinnitus and clinical observations. The causal interrelations between tinnitus distress, perceived loudness and cognitive abilities (specifically executive functions) are the focus of the EDM.

### Conceptual EDM

**Figure 3:**
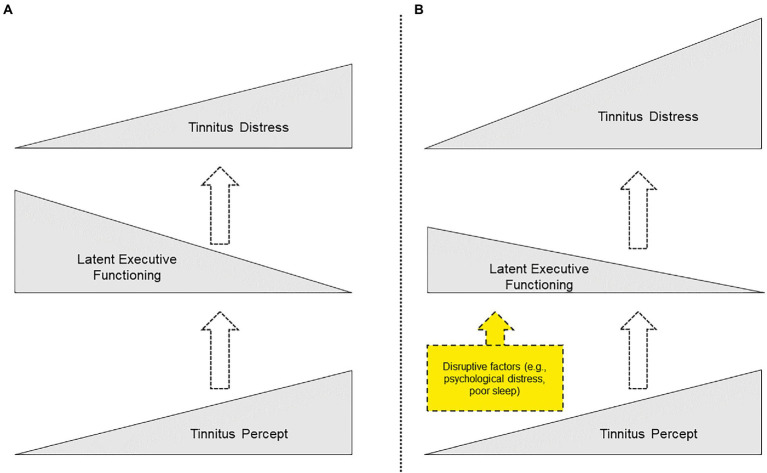
**(A)** Conceptual illustration of EDM. A variable tinnitus percept is combined with variable latent executive functioning to result in tinnitus distress (height of triangles depicts magnitude of each construct and arrows depict tinnitus intrusiveness). **(B)** Conceptual depiction of the impact of disruptive factors (e.g. psychological distress, disrupted sleep, etc.) that reduce latent executive functioning and consequently result in increased tinnitus distress.

The EDM is depicted in Figure 3A. The bottom triangle depicts a tinnitus percept of variable intensity across individuals. The moment-to-moment intrusiveness and resulting distress depends on an individual’s executive functioning (depicted by the middle triangle). An individual’s tinnitus distress level is depicted by the top triangle and is posited to arise from the combination of these two elements. For example, a quiet tinnitus percept that is combined with increased latent executive functioning will diminish the intrusiveness of the tinnitus percept, and subsequently cause minimum tinnitus distress. At the opposite end of the spectrum, a loud tinnitus percept combined with lower executive ability results in maximum tinnitus distress.

The EDM proposes that tinnitus distress is caused by intrusion of the tinnitus percept and subsequent distress resulting from inherent variability and temporary disruption of latent executive abilities. Latent executive abilities are typically well established in people by the time they develop chronic tinnitus. Executive abilities develop throughout life and are non-linear in trajectory; different subskills develop at different ages and have different developmental trajectories (Best et al., 2009; Gilmore and Cragg, 2018). By adulthood, individual differences are generally established in executive abilities (Friedman and Miyake, 2017), potentially predisposing some individuals to find a tinnitus percept more intrusive.

Whatever a person’s individual executive functioning capacity may be, executive functioning may be disrupted by external factors. Diamond (2013) notes that executive abilities are the first cognitive abilities to be compromised by stress and life events. Execution functioning may be disrupted by various factors. Many of these disruptive factors are established correlates of tinnitus distress, such as poor sleep and stress. Figure 3B depicts how this disruptive effect might reduce the protective effect of latent executive functioning, resulting in relatively increased tinnitus distress. Tolerance of the percept (i.e. tinnitus distress) may differ depending on stress levels, depressive mood, anxiety levels or cognitive changes, with a subsequent increased perception of tinnitus intrusiveness and resultant distress.

To provide initial evidence of validity, this study aimed to evaluate the EDM in two independent datasets using the methodology outlined in the remainder of this article.

## Materials and methods

The theoretical underpinnings and conceptual basis of the EDM of tinnitus distress were detailed in the previous section. The following sections describe the datasets, measures, and methods used in this analysis. A description of how the conceptual EDM was translated to a directed acyclic graphic to obtain the minimum adjustment set is then provided. Finally, a two-stage factor score regression analysis using the minimum adjustment set in both datasets is presented.

### Datasets

Two independent datasets were collected. the Construction Dataset was used to develop the model, and the Validation Dataset was used to evaluate the model. Both studies received ethical approval from the University of Nottingham and National Health Service research ethics committees. the Construction Dataset was granted approval by University of Nottingham School of Medicine Research Ethics Committee (Ethics reference number: 83141-1811) and NHS Liverpool East Research Ethics Committee (IRAS project ID: 242476). Data were collected between April 2019 and June 2020. the Validation Dataset was granted approval by the Derby 1 Research Ethics Committee (Reference: 13/EM/0192) on 18 June 2013 and was sponsored by the Nottingham University Hospitals NHS Trust Research and Development (Reference: 13IH001).

In both studies, participants were recruited from both communal and clinical settings to obtain a broadly representative sample of the population with tinnitus. All participants provided informed consent. Both the Construction Dataset1 (*n* = 96) and the Validation Dataset (*n* = 200) included English-speaking participants, aged 18- to 80-year old who experienced tinnitus. the Construction Dataset recruited participants with various levels of tinnitus distress through two groups of 48 participants (THI scores: <38 and ≥38), while the Validation Dataset used stratified sampling to recruit four groups of 50 participants (THI scores: 0–16, 18–36, 38–56 and >58). Table 1 provides an overview of the characteristics of each dataset.

**Table 1:** Descriptive statistics (sample sizes, means and standard deviations) for the primary measures of both datasets used in this study.

	The Construction Dataset	The Validation Dataset
Number of participants	96	200
Age	62.6 (10.2)	59.5 (12.7)
Tinnitus handicap inventory	36.1 (23.8)	35.8 (23.2)
Pure tone audiometry*	20.6 (11.1)	26.0 (19.1)
Psychological distress**	23.6 (18.7)	23.4 (18.1)
Matrix reasoning (WASI-II)	18.8 (4.1)	NA
Keep track task	22.4 (4.4)	NA
Letter number sequencing	20.7 (3.3)	NA
Matrix reasoning (WAIS-IV)	NA	18.6 (4.6)
Wisconsin card sorting task	NA	4.9 (1.7)
Digit span backwards	NA	8.5 (2.8)

*Mean value of thresholds values at 0.5, 1, 2 and 4 kHz to obtain the better-ear-average hearing thresholds. ** The Construction Dataset = DASS21, The Validation Dataset = CORE-OM.

### Inclusion criteria

Both studies required that participants had normal or corrected-to-normal vision, no prior cognitive dysfunction related to issues such as head injury, and no relevant medicinal or recreational drug use. Both also required participants to be able to understand speech in quiet at conversational levels (with or without hearing aids), while the Construction Dataset additionally required participants to have average hearing thresholds of no more than 40 dB HL in the better ear at 500, 1000, 2000 and 4000 Hz (Table 1).

### Study designs

Both studies were prospective, cross-sectional designs where participants completed various self-reported measures of cognition, tinnitus, psychological distress and perceived hearing handicap; various behavioural performance measures were also undertaken that measured executive functioning. Participants underwent pure-tone audiometry (PTA) screening in a sound-treated booth to ensure that hearing thresholds met the study criteria.

The Construction Dataset featured a test battery that took place in a quiet, well-lit room and took approximately 2.5 h to complete. To attenuate order effects such as fatigue and context/contrast effects, a unique sequence of cognitive tasks was assigned to each of the participants. This was achieved through a pseudorandom process in R statistical programming environment, where one of four unique testing sequences was randomly assigned to the participants. All cognitive tasks were performed on a laptop computer. Before completing the executive functioning performance measures, participants sat directly in front of the computer while the investigator confirmed that they were comfortable and could see the written instructions on the screen. Each task featured practise trials; during all practise trials, the investigator sat with the participant to ensure that participants understood task instructions and were comfortable being left to complete the main trials without further supervision.

The Validation Dataset featured a test battery comprising self-reported measures and cognitive performance tasks. It took 2–4 h to complete. Test batteries for each participant were undertaken using one of five different orderings and featured a counterbalanced administration to minimise systematic tiredness or fatigue effects. All testing procedures followed manufacturer/developer instructions.

### Measures

The following section describes the measures used from both datasets that are relevant to the statistics obtained (i.e. factor score regression). A directed acyclic graph based on the EDM was initially developed using the Construction Dataset, which contained variables from a study that investigated differences in various hearing, tinnitus and cognitive measures. For the Construction Dataset, measures (both self-reported and performance-based measures) were chosen by design of the study authors to assess the relationship between tinnitus distress and executive function performance based on a systematic review and meta-analysis on this topic (Clarke et al., 2020). For the Validation Dataset, the measures were chosen based on whether they provided either an identical (e.g. measures of tinnitus distress), or theoretically appropriate match (measures of psychological distress and executive functioning performance). Self-reported measures included cognitive failures, cognitive reserve, rumination and hearing handicap. Items concerning tinnitus laterality and duration, as well as participant age and gender were available in both datasets. A full description of the measures in each study used in this analysis is available in each thesis (Mohamad, 2015; Clarke, 2021). The analysis of directed acyclic graphs presented in this study demonstrated that these measures were not required to estimate the total causal effect of interest; therefore, only measures featuring in the linear modelling analyses are described.

### Hearing thresholds

In both datasets, PTA was performed using an adaptive staircase technique (British Society of Audiology, 2017) in 5 dB HL steps in a sound-attenuating booth. The mean value of thresholds values at 0.5, 1, 2 and 4 kHz was calculated to obtain the better-ear-average hearing thresholds, with higher scores represent poorer hearing thresholds.

### Self-reported measures

#### Tinnitus distress

The Tinnitus Handicap Inventory (THI) features 25 items and was intended to measure individual’s perceived handicap resulting from their tinnitus (Newman et al., 1998). However, various studies of its factor structure have reported the THI to be a unidimensional measure of tinnitus distress, with ‘excellent’ internal consistency (α > 0.9) having been previously reported (Fackrell et al., 2014). Each item features a 3-option ‘Yes’, ‘No’ or ‘Sometimes’ response, with a numerical equivalent of 4, 0 and 2 points scored, respectively. The measure of tinnitus distress is obtained by summing scores and has been assigned five clinical categories (McCombe et al., 2001): Grade 1—slight or no handicap (0–16), Grade 2—mild handicap (18–36), Grade 3—moderate handicap (38–56), Grade 4—severe (58–76) and Grade 5—catastrophic (78–100). A total of 100 can be scored on the THI; larger scores represent increased perceived tinnitus distress.

#### Psychological distress

In the Construction Dataset, the Depression Anxiety and Stress Scales (DASS21) was used to assess general psychological distress; it is a shortened version of the DASS questionnaire that contains 21 items and has psychometric validation evidence in clinical groups and a community sample (Antony et al., 1998); ‘strong internal consistency’ has been reported in previous studies (α > 0.8). It comprises three subscales: Depression (i.e. dysphoric mood such as feelings of sadness or worthlessness), Anxiety (i.e. symptoms of physical arousal such as panic attacks and fear responses) and Stress (i.e. symptoms of tension and irritability such as tendencies to overreact to stressful events). Participants are asked to rate the applicability of example symptoms during the last 7 days (e.g. ‘I found it hard to wind down’ for stress) using a 0–3 scale. Larger scores on the DASS21 represent increased depression, anxiety or stress, with an accompanying categorisation system provided for ‘normal’ (9, 7 and 14-point thresholds, respectively), ‘mild’ (13, 9 and 18-point thresholds, respectively), ‘moderate’ (20, 14 and 25-point thresholds, respectively), ‘severe’ (27, 19 and 33-point thresholds, respectively), and ‘extremely severe’ (28, 20 and 34-point thresholds, respectively).

In the Validation Dataset the Clinical Outcomes in Routine Evaluation-Outcome Measure (CORE-OM) was used to assess psychological distress (Barkham et al., 2006). Evans et al. (2002) described the CORE-OM as having ‘excellent’ internal consistency (α > 0.9). The CORE-OM comprises four subscales: Wellbeing (feelings about self), Problems/symptoms (anxiety or depression), Functioning (functioning on a daily life and relationship with others) and Risk (self-harm or other). The CORE-OM includes 34 items with recall periods spanning ‘over the last week’. Each item features a 5-point response scale from 0 to 4 (0 = not at all, 1 = only occasionally, 2 = sometimes, 3 = often and 4 = most or all the time). The total score was used (ranging from 0 to 136 points) with higher global scores representing a greater problem.

### Executive functioning performance measures

Various executive functioning performance tasks were selected to comprehensively measure executive functioning in each dataset. The measures used in the Construction Dataset were fundamentally informed by the multi-factor framework of executive functioning that views fluid ability as a higher-order executive function and necessitates lower-level executive functions to achieve reasoning, problem solving and planning (Diamond, 2013; Friedman and Miyake, 2017). To attain a unitary factor of executive function from both datasets, measures of fluid ability (i.e. matrix reasoning tasks) were chosen, with appropriate tasks to tap other executive functions (i.e. working memory/updating, shifting/cognitive flexibility and inhibition). The measures chosen align with contemporary view that inhibition is supportive of working memory (Diamond, 2013). Moreover, resulting from task impurity, (Rabbitt, 2004) all measures can be considered to measure executive functioning to some extent; although, some emphasise aspects of a superordinate executive functioning factor, all contain supportive executive functioning components and were ultimately reduced to common variance factor scores of executive functioning through principal axis factoring.

#### Matrix reasoning (WASI-II)

In the Validation Dataset, the matrix reasoning subtest of the Wechsler Abbreviated Scale of Intelligence was selected as a measure of fluid intelligence (Duncan, 2010), it is widely used for clinical and research purposes (Saklofske and Schoenberg, 2011). It features incomplete series of complex shapes, and the participant is required to select a final item (from a total of five items) that best completes the series, with a total of thirty points being available. The task becomes increasingly difficult as the test progresses, and the procedure is terminated when participant answers three consecutive items incorrectly. The total number of correct responses is summed to obtain a score where larger scores demonstrate better performance.

#### Matrix reasoning (WAIS-IV)

In the Construction Dataset, the matrix reasoning task subtest was used from the WAIS-IV (Drozdick et al., 2012) and was chosen as a measure of fluid intelligence (Duncan, 2010). The task is fundamentally the same as that described for the WAIS-II, except a total of 26 points was available. Similarly, participants completed the task after selecting answers or if they selected an incorrect answer on three consecutive series.

#### Wisconsin card sorting task

In the Validation Dataset, the Wisconsin Card Sorting Test (WCST) was chosen as a measure that emphasised shifting/cognitive flexibility. A computerised scoring software was used to eliminate recording and scoring errors (Heaton et al., 1993). This automatically calculated the two measurement variables, including the scaling according to age and educational level. In this version, participants were instructed to match a series of test cards (128 test cards in 2 packs) to four reference cards according to one of three rules (colour, shape or the number of stimuli on the card). The participant was not informed what the matching rule is and must work this out using the feedback (correct or incorrect) given by the experimenter after each trial. The matching rule was changed following 10 consecutive correct responses on a specific matching category. Participants must then work out a new rule using experimenter feedback. The test continues until either the participant has successfully completed six matching rules, or all 128 stimulus cards have been viewed. Various scoring methods have been devised for the WCST. This analysis used the number of matching rules (i.e. each sequence of ten correct responses to a specific matching rule) completed during the test. Scores can range from 0 to 6 with higher scores demonstrating better performance.

#### Digit span backwards

In the Validation Dataset, the Digit Span Backwards was chosen as a measure that emphasises working memory. A subtest of the WAIS was administered using a computer connected to a pair of loudspeakers. The speech was adjusted individually to a comfortable listening level for every participant, who was required to repeat a sequence of digits in reverse order. Sequences started with two digits and featured a maximum of seven. The number of digits increased by one following two trials of the same length until two trials of the same length were failed or the maximum number of digit sequences were correctly repeated. Each correctly repeated sequence was summed and the score is the total of the points from correctly recalled sequences with a maximum score of 14.

#### Keep track task

In the Construction Dataset, the Keep Track Task was chosen as a measure that emphasises updating working memory (Yntema, 1963; Miyake et al., 2000). A computerised version was constructed in PsychoPy (Peirce et al., 2019). This task features six categories (animals, colours, countries, distances, metals and relatives), and participants were randomly assigned categories as ‘target’ categories at the start of each trial, beginning with three and increasing to five categories. During each trial, a sequence of words from all six categories was randomly displayed on the screen for 2 s. Participants were required to recall the last word they saw from their target categories and type them into the computer at the end of each trial. To verify conceptual understanding of this challenging task, participants completed two practise trials with the investigator in the room before completing the main trials with no supervision. Participants completed three trials each with three target categories (3 trials), four target categories (3 trials) and five target categories (3 trials). A total of 36 correct target words could be achieved in this task, with higher scores demonstrating better performance.

#### Letter number sequencing

In the Construction Dataset, the Letter Number Sequencing task was chosen as a measure that emphasises updating of working memory (Diamond, 2013). A computerised implementation using PsychoPy was used that featured stimuli from the subtest of the WAIS-IV. During the task, participants were presented with a stimulus string on a screen that contained a mixture of letters and numbers (e.g. C71). The stimulus was required to be remembered by the participants and reported in the correct sequence, where numbers are reported first in ascending order, followed by letters in alphabetical order (e.g. 17C). The task increased in difficulty as letters and numbers were incrementally added to the stimulus string. This task began with five practise trials featuring stimuli strings of two or three alphanumeric characters; the practise trials familiarised participants with the task and allowed the investigator to check that the task instructions were understood by the participant. The participant then completed 30 experimental trials containing 2–8 alphanumeric characters. A total of 30 correct answers could be obtained in this task, with higher scores demonstrating improved performance.

### Analyses

Directed acyclic graphs were designed and analysed using Dagitty (Textor et al., 2016). Factor analysis for factor scores regression was performed in R using with factor scores generated using the ‘psych’ package (Revelle, 2017).

### Directed acyclic graph analysis to obtain the minimum adjustment set

Directed acyclic graphs are graphical representations of an assumed data-generating process that describe a non-parametric relationship between variables; they provide a simple means of demonstrating theories and assumptions about causal relationships between variables in observational studies, and as such are being increasingly employed in applied healthcare research (Tennant et al., 2021). An attractive feature of directed acyclic graphs is that, through visual inspection or algorithmic identification, they allow researchers to obtain the ‘minimum adjustment set’, which is a minimally sufficient set of covariates that enable the estimation of the total causal effect of interest based on the model.

A directed acyclic graph was created by mapping variables that were relevant to the EDM in the Construction Dataset to a directed acyclic graph structure. the Construction Dataset was chosen because the study that generated it had a specific focus on the relationship between tinnitus distress and executive functioning; therefore, it contained all the theoretically relevant variables to the EDM, as well as those already contained in the extant the Validation Dataset (i.e. the variables available in the Validation Dataset were a subset of those in the Construction Dataset). This approach also meant that multiple directed acyclic graphs could be produced and analysed to understand if those variables considered theoretically relevant to the EDM (i.e. those in the Construction Dataset) were required to estimate the causal effect of tinnitus distress on executive functioning in the Validation Dataset. This was done by creating directed acyclic graphs and comparing the required minimum adjustment sets necessary to estimate the effect of executive functioning on tinnitus distress. The mapping process entailed explicating theoretical relationships between the nodes of the model using a combination of EDM-based theoretical assumptions and inferences drawn from the wider tinnitus literature to construct each directed acyclic graph.

### Factor score regression

Two-stage factor score regression was used to estimate the effect of executive functioning on tinnitus distress in each dataset. Factor score regression is an alternative method to estimate structural equation models in small sample sizes by first obtaining scores for each subject on each factor, which can then be used for further analysis in place of measured variables in a regression model (Odum, 2011). In the first stage, factor scores were estimated using principal axis factoring in both datasets. In the second stage, general linear models were specified using the minimum adjustment set obtained from the directed acyclic graph analysis of the EDM structural equation metamodel.

Principal axis factoring is a data reduction technique that extracts common variance from variables to obtain a measure of latent executive functioning. Like all factor analytic techniques, it generally requires appropriately large sample sizes to undertake; while general sample size guidelines are available, the appropriateness of factor analysis depends on the specific datasets to which it is applied. Due to the relatively small sample size available in the Construction Dataset, the suitability of both datasets for factor analysis was first investigated before calculating factor scores. The Kaiser-Meyer-Olkin (KMO) index was calculated to assess the appropriateness of applying principal axis factoring to both datasets. This provides a measure of sampling adequacy ranging from 0–1 where values <0.5 are considered unacceptable (Kaiser, 1974). Principal axis factoring appropriateness was also assessed with Bartlett’s test of sphericity, which tests the sufficiency of correlations in a dataset as a perquisite for factor analysis.

Matsunaga (2010) notes that ‘latent factors should be determined primarily on the grounds of theoretical expectations and conceptualisations of the target construct’. In this instance, a single factor relating to executive functioning was conceptualised and expected. However, many procedures have been created to provide indication of adequacy of a factor solution. Several of these were implemented to provide support for the appropriateness of a single factor solution; these included eigenvalues, scree tests and parallel analysis (Revelle, 2013). Eigenvalues measure the amount of variation in the total sample accounted for by each factor. The Kaiser-Guttman criteria suggest that eigenvalues exceeding 1 are viable factors and should be retained, as they explain more variance within the data than a single variable. Scree tests plot successive eigenvalues and look for a sudden drop in eigenvalues or ‘break’ in the plot, which indicates the appropriate number of factors. Parallel analysis extracts factors until the eigenvalues of the real data are less than those of a random dataset of the same size.

Factors scores were subsequently calculated to use as predictor variables in general linear models to estimate the effect of executive functioning on tinnitus distress. When estimating causal effects using directed acyclic graphs and linear models, predictor variables (or covariates) should not be included without sufficient justification as they can confound parameter estimates (McElreath, 2016). Therefore, the linear models were specified using the minimum adjustment set identified in the directed acyclic graph analysis of the EDM to obtain unbiased parameter estimates of the effect of executive functioning on tinnitus distress in both datasets. Various factor scoring methods exist, including regression or ‘Thurstone’ scores, ‘Bartlett’ scores and Anderson-Rubin scores, with each providing slightly different estimates of factor scores (Distefano et al., 2008; Revelle, 2013). When used in factor score regression, different factor score methods may produce biased parameters estimates; however, unbiased/corrected estimates can be obtained through various approaches, including the use of the Thurstone scoring method when factor scores are the predictor variables in linear models (Skrondal and Laake, 2001; Devlieger and Rosseel, 2017). There is no consensus regarding the optimal method for obtaining factor scores (van de Schoot and Miočević, 2020), therefore, as a sensitivity analysis, factor scores were computed using both Thurstone and Bartlett scoring methods to be used in the linear models.

The following sections describe the construction and subsequent causal analysis of directed acyclic graphs based on the EDM to obtain the minimum adjustment set, which was subsequently used to estimate the effect of executive functioning on tinnitus distress using factor score regression.

### Constructing causal models of the EDM

**Figure 4:**
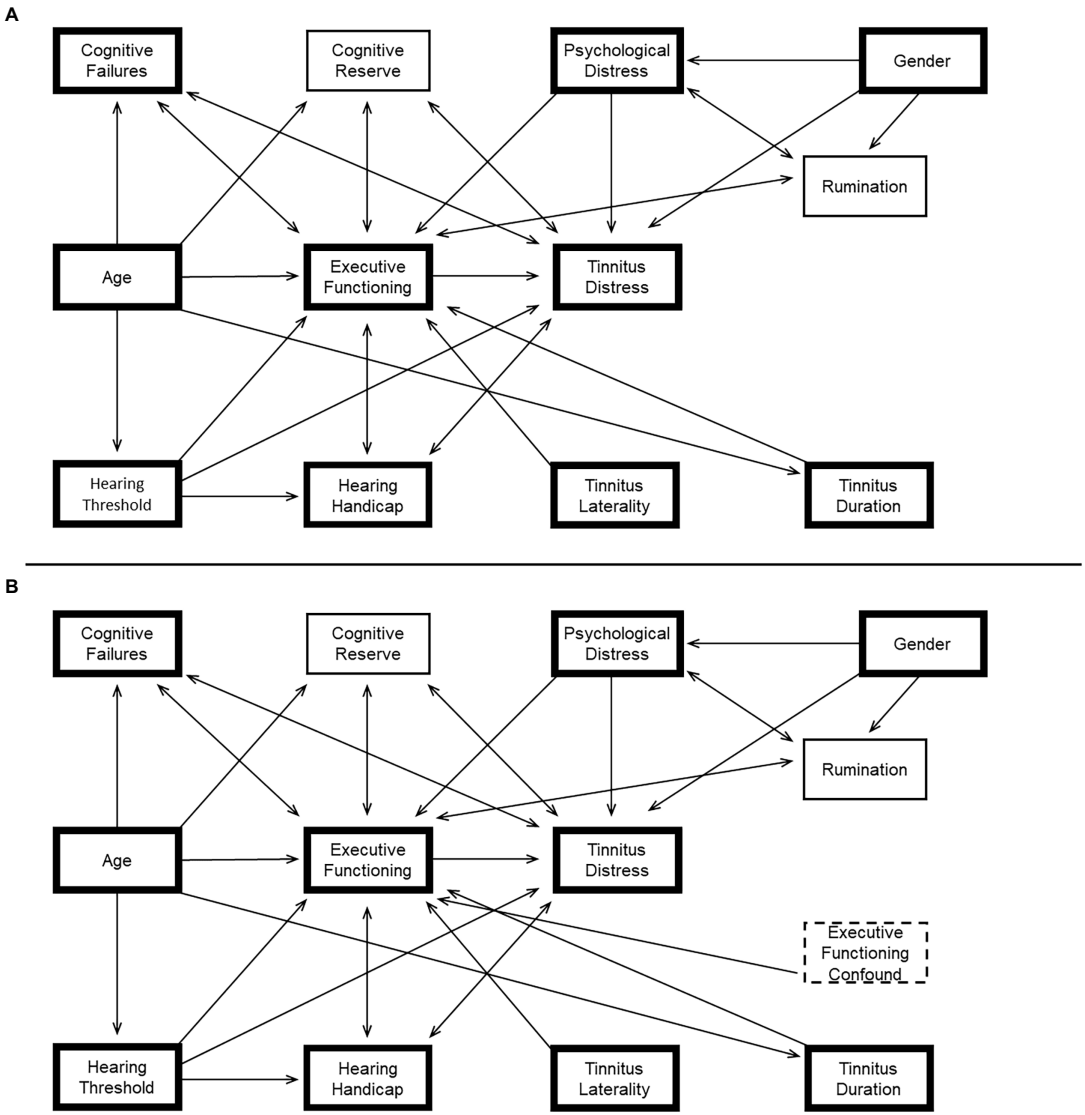
**(A)** The conceptual EDM of tinnitus distress was translated into a directed acyclic graph. Bold boxes show the measures that were available in both datasets. **(B)** Shows the same directed acyclic graph with an additional unmeasured confounding node (e.g. tinnitus loudness) causally impacting executive functioning. The minimum adjustment set required to estimate the total causal effect of Executive Functioning on Tinnitus Distress is equivalent in all configurations of the directed acyclic graph (i.e. Minimum Adjustment Set = {Psychological Distress, Best-Ear Averaged Hearing Threshold}).

As the EDM is a conceptual model, it is mapped to a causal model (i.e. a directed acyclic graph), which provides a rigorous framework for addressing causality in complex models; the directed acyclic graph presented in Figure 4A represents assumptions regarding the relationship between executive functioning performance and tinnitus distress. Nodes within the model are specified based on logical causal relationships, insights from empirical research and theoretical interrelations informed by the wider literature. Code for the directed acyclic graphs described in Figure 4 is provided in an appendix.

Logical causal relationships are primarily nodes on the periphery of the model and include relationships between age, cognition and hearing thresholds. For example, while there is a well-known association between age and hearing thresholds, the causal relationship can be confidently specified with a unidirectional arrow (Age → Hearing Threshold) because hearing thresholds can never be the cause of a person’s chronological age; neither can their self-perceived cognitive failures or executive abilities. Similarly, an individual’s level of tinnitus distress cannot cause their gender, duration of tinnitus or perceived tinnitus laterality. Hearing handicap cannot cause hearing thresholds. Psychological distress or rumination cannot cause self-reported gender.

Various paths have been specified based on research and theoretical assumptions. In Clarke et al. we demonstrated an association between executive functioning and tinnitus distress through meta-analysis. The EDM suggests a causal relationship, with executive abilities causing tinnitus distress. We also demonstrated an association between tinnitus distress and self-perceived cognitive failures. However, the causal relationships between cognitive reserve, cognitive failures, rumination and executive functioning are not readily inferred and cannot be confidently specified in the model (e.g. people could perceive more cognitive failures because of poorer executive functioning, or an expectation of failures may impact executive functioning). In these instances, the model is agnostic about plausible but uncertain causal relationships, and simply specifies an association with a bidirectional arrow.

Theoretical interrelations for causal and associative relationships between various nodes are also drawn from the wider literature. Associations have been reported between rumination and executive function (Yang et al., 2017), as well as rumination and general psychological distress (Michl et al., 2013; Wilkinson et al., 2013). Gender differences in rumination have been reported (Johnson and Whisman, 2013), and a causal relationship could be specified because rumination cannot cause gender. Associations have been reported between hearing handicap and tinnitus distress (Henry, 2016; Ivansic et al., 2017); as the causal relationship between them is unknown, an association is specified. Executive function and hearing handicap share an association (Lee et al., 2019). Cuny et al. (2004) have suggested that the laterality of a tinnitus percept may direct attention towards the tinnitus sound; however, executive functioning cannot cause tinnitus laterality, a causal relationship could be specified. Diamond (2013) notes that executive functions are the first cognitive functions to ‘suffer and suffer disproportionately’ due to psychological distress; a causal relationship is therefore specified. Finally, an individual’s tinnitus duration may feasibly impact their tinnitus distress through executive functioning because habituation to the stimulus may lessen tinnitus distress over time; therefore, a causal relationship between these variables can be specified.

## Results

### Obtaining the minimum adjustment set from EDM causal models

Including every measured covariate within a statistical model may confound causal estimates (McElreath, 2016). Path analysis of directed acyclic graphs informs researchers of necessary variables to obtain a conditional causal estimate of interest in proposed models. The required variables are collectively known as the minimum adjustment set.

Having specified the directed acyclic graph, path analysis can be algorithmically applied to provide the minimum adjustment set for the designated path to provide a causal estimate. Failing this, the causal path analysis also importantly indicates where no estimate is possible based on causal structure specified in the directed acyclic graph. Informally, the directed acyclic graph analysis to obtain the minimum sufficient adjustment set works by identifying a set of variables that would block all biasing paths in a given causal diagram (Acid and De Campos, 2013).

The direct causal path of interest (i.e. the total effect of executive functioning on tinnitus distress) is shown in Figure 4A. Causal path analysis of the directed acyclic graph shows that there is only one minimum adjustment set based on this model, where the total effect of executive functioning on tinnitus distress can be estimated after correcting for psychological distress, and hearing thresholds (i.e. minimum adjustment set = {Psychological Distress, Best-Ear Averaged Hearing Threshold}). It is noteworthy that age is absent from the minimum adjustment set in this model, and unnecessary inclusion of this covariate within a regression model could confound the resulting parameter estimates.

An important feature of causal path analysis with directed acyclic graphs is that they allow sensitivity and robustness checks to be performed on modelling assumptions; this may be done through adding or removing nodes and paths to demonstrate how the resulting model would impact the ability to estimate a causal effect. For example, in the context of the EDM, tinnitus loudness and disrupted sleep could both be considered important variables affecting cognitive performance. A directed acyclic graph with unmeasured confounds (Executive Functioning Confound) was also created to assess the impact of their inclusion on the EDM-directed acyclic graph (Figure 4B). The minimum adjustment set provided by Figure 4B shows that, even with the addition of an unmeasured executive functioning confound (e.g. disrupted sleep), the estimate of the total causal effect of Executive Functioning on Tinnitus Distress can still be performed with the same minimum adjustment set (i.e. Minimum Adjustment Set = {Psychological Distress, Best-Ear Averaged Hearing Threshold}).

Figures 4A,B shows the variables that were available in both datasets (i.e. the subset of variables from the directed acyclic graph based on the Construction Dataset, used to develop the EDM); Appendix A provides the code for each directed acyclic graph used to produce the minimum adjustment sets relevant to variables contained in both the Construction Dataset and the Validation Dataset. The minimum adjustment set was also analysed for a directed acyclic graph consisting only of these variables. The causal path analysis showed that the minimum adjustment set to estimate the total effect of executive functioning on tinnitus distress considering only these variables was equivalent (i.e. minimum adjustment set = {Psychological Distress, Best-Ear Averaged Hearing Threshold}).

### Validating the EDM causal model

#### Obtaining latent measures of executive functioning using principal axis factoring

The appropriateness of performing factor analysis in both datasets was evaluated and confirmed. Bartlett’s test of sphericity was statistically significant in both (*p* < 0.001) and measures of sample adequacy were acceptable (>0.6). Principal axis factoring was subsequently applied to measures of executive functioning in each dataset using three theoretically comparable executive functioning performance measures from each dataset to obtain a latent measure of executive functioning. In the Construction Dataset, these were the Matrix Reasoning, Letter Number Sequencing and Keep Track Tasks. In the Validation Dataset, the measures were the Matrix Reasoning, Wisconsin Card Sorting Task and the Digit Span Backwards subset of the WAIS-IV. These measures were identified as most appropriate for external validation due to their theoretical similarity and overlap for representing executive functioning across the datasets.

A single latent factor of executive functioning was supported with factor loadings for all measures exceeding a minimum cut-off value of 0.4 (Matsunaga, 2010) in both datasets. Eigenvalues for both datasets exceeded the Kaiser criteria, and both scree plots and parallel analysis supported the theoretical expectation and appropriateness of a single latent factor solution (not shown). A single factor principal axis factor was specified (the Construction Dataset principal axis factor: Matrix Reasoning = 0.7, Keep Track Task = 0.5, Letter Number Sequencing = 0.6 and the Validation Dataset principal axis factor: Matrix Reasoning = 0.8, WCST = 0.5, Digit Span Backwards = 0.5).

#### Factor score regression with the minimum adjustment set

To validate the central tenet of the EDM, factor score regression was subsequently performed to estimate the causal total effect of executive functioning on tinnitus distress in both datasets using the minimum adjustment set obtained from the EDM-directed acyclic graph analysis (i.e. {Psychological Distress, Best-Ear Averaged Hearing Threshold}). Therefore, a general linear model was specified for each dataset with tinnitus distress as a response variable, and executive functioning factor scores, averaged hearing thresholds and psychological distress scores as predictor variables. As a sensitivity check, factor scores were computed with both Thurstone and Bartlett scoring methods, which yielded no substantive difference to the estimated parameters; therefore, factor score regressions with Thurstone factor scores are reported in line with recommendations in Skrondal and Laake (2001).

The assumptions of the linear regression analyses were assessed in both datasets: linearity was assessed (*via* assessment of plotted residuals and fitted values), homogeneity of variance (*via* plot of square root of standardised residuals), excessively influential observations (*via* assessing a plot of leverage and standardised residuals), collinearity of predictors (*via* variance inflation factors) and normality of residuals (*via* a Q-Q plot). Assumptions were met according to typical thresholds in both datasets, with the exception of homogeneity of variance, notably in the upper and lower ranges of fitted values of each dataset (i.e. both models displayed heteroskedasticity); however, heteroskedasticity was not considered problematic because the primary objective was to obtain parameter estimates of the effect of executive functioning on tinnitus distress, and ordinary least squares is known to provide unbiased parameter estimates in the presence of heteroskedasticity (Hayes and Cai, 2007). The reliability of the outcome variable (tinnitus distress) was evaluated in both datasets using Cronbach’s alpha (the Construction Dataset = 0.76, the Validation Dataset = 0.94), and found to be above the typically acceptable thresholds in both datasets (Nunnally and Bernstein, 1993).

The linear model parameter estimates for factor scores were highly congruent in both datasets (Table 2). The parameter estimate for executive functioning factor scores in the Construction Dataset was−3.50 (*p* = 0.13), while the parameter estimate for executive functioning factor scores in the Validation Dataset was−3.71 (*p* = 0.02). The linear models show that executive functioning is negatively associated with tinnitus distress to a similar degree (i.e. as executive functioning performance increases, self-reported tinnitus distress decreases by the same amount in both datasets).

**Table 2:** Factor score regression results for the Construction Dataset (column 1) and the Validation Dataset (column 2).

Dataset	Estimate		Standard error		95% Confidence intervals		Statistic (Pr > t)		*p*-value		*R* ^2^	
	1	2	1	2	1	2	1	2	1	2	1	2
											0.46	0.38
Intercept	36.06	35.81	1.81	1.30	32.46, 39.66	33.25, 38.37	19.88	27.61	<0.01	<0.01		
Executive Functioning Factor Scores	**-3.50**	**-3.71**	2.31	1.59	−8.10, 0.99	−6.87, −0.55	−1.51	−2.35	0.13	0.02		
Hearing Threshold	−2.69	0.42	1.90	1.32	−6.46, 1.09	−2.19, 3.03	−1.41	0.33	0.16	0.74		
Psychological Distress	14.95	13.59	1.88	1.31	11.21, 18.69	11.01, 16.19	7.94	10.34	<0.01	<0.01		

Highlighted in bold are the parameter estimates of the total causal effect of executive functioning on tinnitus distress, which is nearly identical.

Although *p*-values are provided in this study for consistency with contemporary analytic practises, our primary goal was parameter estimation of factor scores across independent datasets; therefore, interpretation of these results through a statistical hypothesis testing framework at the conventional level was not undertaken (i.e. *p* < 0.05). Interpretation was based on previous research demonstrating associations between tinnitus distress and executive functioning (Clarke et al., 2020).

## Discussion

This analysis provides initial validation of the EDM of tinnitus distress by using factor score regression to produce essentially identical parameter estimates of the total causal effect of executive functioning on tinnitus distress in two independent datasets. By using the same theoretical model and confirmed minimum adjustment set in independent datasets, combined with different measures to extract a latent measure of executive functioning, this study demonstrates that as executive functioning increases, tinnitus distress decreases. The results are supported with a clear underpinning theory and conceptual model of the role of executive functioning in tinnitus distress.

When considering tinnitus distress and cognitive performance, causality is a crucial consideration because researchers frequently default to causal language. This mode of thinking and associated language frequently belies a widespread, yet tacit assumption that the tinnitus percept causes poorer cognitive performance; this is demonstrated through the causal language that researchers frequently default to when describing the relation between tinnitus and cognition. Examples include descriptions such as, ‘the impact of tinnitus severity on cognition’, “tinnitus and its effect on working memory and attention’ and ‘the impact of tinnitus upon cognition’ (Rossiter et al., 2006; Mohamad et al., 2016; Tegg-Quinn et al., 2016). Application of a rigorous causal framework facilitates communication concerning theoretical assumptions that can advance knowledge of the relation between relevant variables. To date, few studies have attempted to develop causal models of tinnitus distress (Cima et al., 2011; McKenna et al., 2014; Handscomb et al., 2019). A strength of this study is the novel incorporation of directed acyclic graphs combined with latent variable methods, informed by a theoretical model, to establish the ability to provide an estimate of interest in independent datasets. This methodology provides researchers with a precise and easily communicated framework to investigate aspects of models such as the EDM across independent datasets.

Although the EDM suggests that the tinnitus percept louder in some individuals (or have some other perceptual quality that contributes independently to tinnitus distress) this analysis showed that inclusion of tinnitus loudness in the minimum adjustment set was not unnecessary to estimate the total causal effect of executive functioning on tinnitus distress; in fact, inclusion of such unnecessary variables may bias parameter estimates of interest (McElreath, 2016). Moreover, a key strength of this study was estimation of the effect of interest across independent datasets that included different measures and study designs.

This analysis was built on previous research with a primary focus on cognitive performance and executive functioning. Although psychological distress is featured in the minimum adjustment set of the estimation of the effect of executive function on tinnitus distress, the focus of the EDM is executive functioning. The parameters from variables in the minimum adjustment set identified in this study were not subject to subsequent interpretation because they were not the parameters of interest, and different minimum adjustment sets would be implied to define specific causal paths. Nonetheless, the EDM is consistent with the possibility for other important contributory factors that have important roles in causing distressing tinnitus such as psychological distress. Moreover, other studies have reported on the relation between tinnitus distress and psychological distress more broadly (Cima et al., 2011; Handscomb et al., 2019), nonetheless, they are also theories and further work is required using optimised study designs to provide validity evidence.

### Clinical implications

Appropriate counselling of tinnitus patients is becoming increasingly recognised as an important clinical task (Beukes et al., 2017; Liu et al., 2018; Taylor et al., 2020). The EDM provides a clear conceptual explanatory framework concerning the relation between tinnitus distress and executive functioning that can readily be adapted for clinical practice. Current clinical advice is informed by models that propose tinnitus distress arises through persistent active monitoring of the percept and subsequent negative thoughts or fear avoidance; to someone seeking advice or assistance, such descriptions may not be consistent with their experience of intrusive tinnitus that simply ‘appears’ without active monitoring. The clinical narrative offered by the EDM does not suggest that people with tinnitus are continually focussing/paying attention to the percept; instead, it proposes that tinnitus distress can arise from disruption to executive functioning through external stressors, which increase the intrusion of the tinnitus percept. Therefore, it may be a more accurate and readily accepted conception for people with distressing tinnitus.

The EDM also suggests the possibility of intervention. Diamond and Ling (2016, 43) argue that executive functioning can be improved ‘at every age from infants through elders and *via* diverse approaches.’ An underexplored question within the tinnitus literature is: what are the benefits of executive function interventions for people with tinnitus? The EDM suggests that such interventions will not be effective for individuals who do not find their tinnitus distressing (i.e. they will have no effect on the tinnitus percept *per se*). However, for people with distressing tinnitus they may be beneficial. The EDM suggests that effective intervention on executive functions (or the causes that disrupt them) may reduce the intrusiveness of the tinnitus percept and therefore improve subsequent tinnitus distress. Various methods aimed at achieving this effect remain to be explored, which include pharmacological products that affect cognitive and executive functioning, non-pharmacological interventions such as cognitive training programs, and indirect interventions that improve executive functioning as a by-product of quality of life-related interventions and general wellbeing programs (Diamond and Ling, 2016).

### Future research

The causal relations considered alongside the EDM are important to understand, as they would inform the likelihood of success of various therapeutic interventions. For example, whether inherent poorer executive abilities are the cause may imply cognitive training is required, whereas disruption through stress would imply that removal or reduction of the disrupting stressor is required. The EDM also suggests that through removing or reducing disruption to the executive abilities (e.g. through reducing global psychological distress or improving sleep) it should be possible to reduce tinnitus distress.

The EDM also entails theoretical predictions. For example, people with louder tinnitus (as measured using psychoacoustic methods) and better executive performance should not find their tinnitus distressing, while those with quieter tinnitus and poorer executive abilities should find their tinnitus equivalently or even more distressing. Longitudinal study designs could also be employed to test the EDM at tinnitus clinics, where a battery of cognitive tasks, measuring latent executive ability are given to people who have recently developed tinnitus. The EDM suggests that the latent measure of executive performance on such tasks will go on to predict levels of tinnitus distress should their tinnitus become chronic. Both predictions can be tested in future research studies as a way of validating the EDM perspective.

### Study strengths and limitations

This modelling work was robust in being able to provide an estimate of the latent executive ability in each dataset using common variance across multiple executive functioning measures. Although these measures were adequate to operationalise executive functioning in each dataset, they were not theoretically optimal and could only represent broad executive functioning. *A priori* selection of measures to measure distinct factors of executive functioning could enable analysis of the relation between tinnitus distress and specific executive functions; for example, a researcher interested in investigating specific aspects of inhibition may follow a similar paradigm used to this study with specifically chosen measures to provide an appropriate latent measure of this aspect of executive functioning.

A limitation of this study is that evidence for validation of the EDM is provided based on point estimates of regression parameters (i.e., the Construction Dataset = −3.50 and the Validation Dataset = −3.71), with the 95% confidence intervals for these parameters being relatively large. This is to be expected given the relatively small sample sizes in both datasets to facilitate latent variable modelling work. Nonetheless, the estimated regression coefficients represent the most likely values of the models given the data, which is remarkably congruent across independent datasets. Moreover, each parameter estimate was fundamentally derived based on a strong theoretical basis, providing a foundation for scientific inference more cogent than would be obtained based on a claim of statistical significance alone (i.e. the existence of non-zero parameter estimates in both samples). It should also be noted that the Validation Dataset provided 95% confidence intervals for the parameter estimate that were non-zero, increasing the confidence that the causal effect derived from theory and estimated from the Construction Dataset is transferable to the general tinnitus population, with a larger sample providing more accurate non-zero parameter estimates of the theoretically predicted effect of executive functioning on tinnitus distress (as would be expected when increasing the sample size alone).

An additional study limitation stems from the partial overlap of variables that were available between the two datasets; due to the retrospective nature of the study design, we were only able to examine a limited part of the proposed conceptual model. However, by using a validated and replicable methodology, this study provided a consistent result across independent datasets. The flexibility and reproducibility of the methods employed enable this causal model to be transferred to new datasets for future studies.

Finally, given the observational nature of the data, a limitation of this study is that the results and subsequent causal interpretation are conditional on the assumptions that have been outlined through the use of structural causal modelling to operationalise the conceptual model of the EDM; and it remains to be seen whether alternative theoretical models can provide a similarly consistent account of the data across independent studies; nonetheless, a key strength of this study is the superordinate theoretical grounding, and explication of the theoretical assumptions in a novel manner that can be applied in future research.

## Conclusion

The EDM is a conceptual model of tinnitus distress that provides a causal explanation for the relation between tinnitus, cognitive performance and tinnitus-related distress. The EDM has been initially validated by using a causal modelling framework to estimate the total effect of executive functioning on tinnitus distress in two independent datasets, which provided essentially identical estimates. The EDM provides fundamental theoretical insights into the relation between tinnitus and cognition and suggests new directions for theoretical development and clinical intervention to reduce tinnitus-related distress.

## Data availability statement

The raw data supporting the conclusions of this article will be made available by the authors, without undue reservation.

## Ethics statement

The studies involving human participants were reviewed and approved by University of Nottingham School of Medicine Research Ethics Committee (ethics reference number: 83141-1811) and NHS Liverpool East Research Ethics Committee (IRAS project ID: 242476). The patients/participants provided their written informed consent to participate in this study.

## Author contributions

NC conceptualisation, data acquisition, methodology, formal analysis and writing (original draft, review and editing). MA conceptualisation, methodology and writing (review and editing). HH conceptualisation, methodology and writing (review and editing). WM data acquisition and writing (review and editing). DHa data acquisition and writing (review and editing). DHo conceptualisation, methodology and writing (review and editing). All authors contributed to the article and approved the submitted version.

## Funding

NC was supported by a Medical Research Council studentship to the former Medical Research Council Institute of Hearing Research. MA is supported by the Medical Research Council (grant number MR/S002898/1). This article reports independent research supported by the National Institute for Health and Care Research (NIHR) Biomedical Research Centre Funding Programme (BRC-1215-20003). The views expressed in this article are those of the author(s) and not necessarily those of the NHS, the NIHR, or the Department of Health and Social Care. (DHo & HH).

## Conflict of interest

The authors declare that the research was conducted in the absence of any commercial or financial relationships that could be construed as a potential conflict of interest.

## Publisher’s note

All claims expressed in this article are solely those of the authors and do not necessarily represent those of their affiliated organizations, or those of the publisher, the editors and the reviewers. Any product that may be evaluated in this article, or claim that may be made by its manufacturer, is not guaranteed or endorsed by the publisher.
